# Hypomagnesemia Is a Risk Factor for Infections after Kidney Transplantation: A Retrospective Cohort Analysis

**DOI:** 10.3390/nu13041296

**Published:** 2021-04-14

**Authors:** Balazs Odler, Andras T. Deak, Gudrun Pregartner, Regina Riedl, Jasmin Bozic, Christian Trummer, Anna Prenner, Lukas Söllinger, Marcell Krall, Lukas Höflechner, Carina Hebesberger, Matias S. Boxler, Andrea Berghold, Peter Schemmer, Stefan Pilz, Alexander R. Rosenkranz

**Affiliations:** 1 Division of Nephrology, Department of Internal Medicine, Medical University of Graz, A-8036 Graz, Austria; balazs.odler@medunigraz.at (B.O.); andras.deak@medunigraz.at (A.T.D.); jasmin.bozic@stud.medunigraz.at (J.B.); anna.prenner@medunigraz.at (A.P.); lukas.soellinger@stud.medunigraz.at (L.S.); marcell.krall@medunigraz.at (M.K.); lukas.hoeflechner@klinikum-graz.at (L.H.); carina.hebesberger@medunigraz.at (C.H.); matias.boxle@medunigraz.at (M.S.B.); 2 Transplant Center Graz, Medical University of Graz, A-8036 Graz, Austria; peter.schemmer@medunigraz.at; 3 Institute of Medical Informatics, Statistics and Documentation, Medical University of Graz, A-8036 Graz, Austria; gudrun.pregartner@medunigraz.at (G.P.); regina.riedl@medunigraz.at (R.R.); andrea.berghold@medunigraz.at (A.B.); 4 Division of Endocrinology and Diabetology, Department of Internal Medicine, Medical University of Graz, A-8036 Graz, Austria; christian.trummer@medunigraz.at (C.T.); stefan.pilz@medunigraz.at (S.P.); 5 General, Visceral and Transplant Surgery, Department of Surgery, Medical University of Graz, A-8036 Graz, Austria

**Keywords:** magnesium, kidney transplantation, infection, urinary tract infection, viral, incidence, immunosuppression, immunity

## Abstract

Introduction: Magnesium (Mg^2+^) deficiency is a common finding in the early phase after kidney transplantation (KT) and has been linked to immune dysfunction and infections. Data on the association of hypomagnesemia and the rate of infections in kidney transplant recipients (KTRs) are sparse. Methods: We conducted a single-center retrospective cohort study of KTRs transplanted between 2005 and 2015. Laboratory data, including serum Mg^2+^ (median time of the Mg^2+^ measurement from KT: 29 days), rate of infections including mainly urinary tract infections (UTI), and common transplant-related viral infections (CMV, polyoma, EBV) in the early phase after KT were recorded. The primary outcome was the incidence of infections within one year after KT, while secondary outcomes were hospitalization due to infection, incidence rates of long-term (up to two years) infections, and all-cause mortality. Results: We enrolled 376 KTRs of whom 229 patients (60.9%) suffered from Mg^2+^ deficiency defined as a serum Mg^2+^ < 0.7 mmol/L. A significantly higher incidence rate of UTIs and viral infections was observed in patients with versus without Mg^2+^ deficiency during the first year after KT (58.5% vs. 47.6%, *p =* 0.039 and 69.9% vs. 51.7%, *p* < 0.001). After adjustment for potential confounders, serum Mg^2+^ deficiency remained an independent predictor of both UTIs and viral infections (odds ratio (OR): 1.73, 95% CI: 1.04–2.86, *p* = 0.035 and OR: 2.05, 95% CI: 1.23–3.41, *p* = 0.006). No group differences according to Mg^2+^ status in hospitalizations due to infections and infection incidence rates in the 12–24 months post-transplant were observed. In the Cox regression analysis, Mg^2+^ deficiency was not significantly associated with all-cause mortality (HR: 1.15, 95% CI: 0.70–1.89, *p* = 0.577). Conclusions: KTRs suffering from Mg^2+^ deficiency are at increased risk of UTIs and viral infections in the first year after KT. Interventional studies investigating the effect of Mg^2+^ supplementation on Mg^2+^ deficiency and viral infections in KTRs are needed.

## 1. Introduction

Magnesium (Mg^2+^) is an essential trace element and the second most abundant intracellular mineral in the body [1]. It exerts several crucial functions in the human body, including actions as a cofactor in cell proliferation and cellular energy metabolism, and it serves as a cofactor of many enzymatic processes [2,3,4].

Accumulating evidence suggests a possible link between Mg^2+^ and the immune system. Mg^2+^ plays an essential role in controlling immune function by exerting an extended effect on several immune processes, such as immunoglobulin synthesis, immune cell adherence, antibody-dependent cytolysis, and regulation of Th1/Th2 responses [5,6]. Recent studies on different Mg^2+^ permeable ion channels and transporters provided new insights into the role of Mg^2+^ in immune responses [7]. These findings led to the discovery that mutations in Mg^2+^ transport systems are the underlying cause of a mild form of combined immune deficiency (CID) named X-linked immunodeficiency with magnesium defect (XMEN) [8], thus supporting the notion that Mg^2+^ signaling is critical for natural killer (NK) and CD8^+^ T-cell function.

Among kidney transplant recipients (KTRs), a high incidence of hypomagnesemia is observed, which seems to be related to the widespread use of calcineurin inhibitors (CNIs), and especially tacrolimus [9]. This phenomenon can mainly be explained by CNI-induced downregulation of transport proteins in the renal tubules leading to renal Mg^2+^ excretion and wasting [10,11,12,13]. The net state of immunosuppression, including induction, maintenance, and anti-rejection therapies, is mainly recognized to increase susceptibility to infections and malignancies, some of which are infection triggered [14].

Opportunistic infections are the most critical complications and a significant cause of graft loss and mortality in KTRs [15,16]. In dialysis patients, hypomagnesemia was associated with an increased risk of death due to infection [17]. In XMEN, Mg^2+^ supplementation increases intracellular Mg^2+^ and normalizes Epstein–Barr virus (EBV) cellular immune response, thus leading to effective viral load suppression [18]. In KTRs, a lower serum Mg^2+^ concentration was associated with an increased risk of severe infections [19]. Nevertheless, data on the association between mild transplant-related infections and Mg^2+^ levels in KTRs in the daily routine practice in the transplant out- and inpatient setting are partly lacking. Knowledge gained from such data may potentially guide our clinical practice on how to deal with diagnosing and treating Mg^2+^ deficiency in KTRs.

Considering the limited data on the relationship between infections after kidney transplantation (KT) and hypomagnesemia, we aimed to examine this association in a cohort of KTRs using retrospective data from a single-center transplant database.

## 2. Materials and Methods

### 2.1. Study Design and Patients’ Characteristics

**Figure 1:**
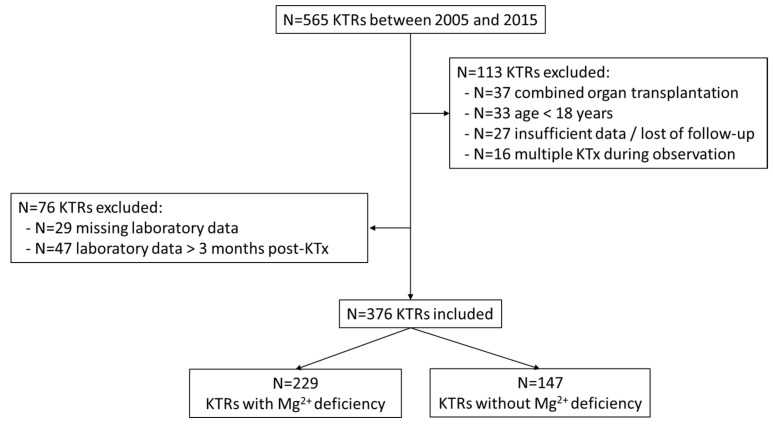
Selection of study population with concomitant serum Mg^2+^ and 25(OH)-vitamin D measurements. (KTR: kidney transplant recipient; KTx: kidney transplantation; Mg^2+^: magnesium).

We conducted a single-center retrospective cohort study of 376 consecutive KTRs transplanted at the Transplant Center Graz (Medical University of Graz) from 1 January 2005 to 31 December 2015. Patients with concomitant serum Mg^2+^ and 25(OH)-vitamin D measurement within three months after KT were identified using our database as published previously (Figure 1) [20].

Male and female patients aged above 18 years were eligible for inclusion in the study if: (1) they received at least one kidney allograft, (2) had a serum Mg^2+^ and 25(OH)-vitamin D measurement within 3 months after KT. Patients were excluded if age was <18 years, they had combined organ transplantation (e.g., pancreas–kidney), or KT follow-up took place at another center. Data were collected after last KT if the patient had more than one KT during the observation period. Data on baseline patient characteristics, primary renal disease, comorbidities, renal replacement therapy (RRT), cytomegalovirus (CMV)-status, transplantation-related data, as well as data on delayed graft function and laboratory findings were collected. Induction (IL2-receptor antagonists (IL-2Ra) or anti-thymocyte globulin (ATG)), maintenance immunosuppression (CNIs, antiproliferative agents (including mycophenolate mofetil (MMF), mycophenolic acid (MPA) and azathioprine (AZA)) or mTOR inhibitors (mTORi—sirolimus, everolimus)) therapies were also recorded. All patients received a standardized corticosteroid therapy with an initial dose of 500 mg prednisolone at the day of transplantation and subsequent tapering to 5 mg per day at month three after KT.

All data were derived retrospectively from the KT and electronic medical records of our center and the Austria Dialysis and Transplantation Registry (OEDTR), as stated previously [20]. The date of KT (index date) was registered for the calculation of time to outcome event. As stated previously, in more than 98% of cases, the ethnicity of the patients was Caucasian, representing the Austrian ethnical background, and was not further specified [20].

### 2.2. Type of Infections, Laboratory, and Clinical Definitions

Data on the most common transplant-related infections, namely urinary tract infections (UTIs) and viral infections, including CMV, polyomavirus (polyoma), and Epstein–Barr virus (EBV), were recorded from 0–12 and 12–24 months after KT. Severe infections requiring hospitalization, defined by the principal diagnosis in the physician’s letter after discharge from the hospital, were also recorded. The diagnostic criteria of UTI in the study were the presence of a positive urine culture (≥10^5^ cfu/mL). CMV, polyoma, and EBV quantitation were done using a real-time polymerase chain reaction (rtPCR) procedure validated by the Diagnostic and Research Institute of Hygiene, Microbiology and Environmental Medicine at the Medical University of Graz. Additionally, if PCR was not available, CMV infections identified by the pp65 antigenemia assay method were also collected.

Serum Mg^2+^ concentrations were measured and validated in the Laboratory Medicine Institute of the Medical University of Graz (normal range of 0.7 to 1.10 mmol/L). Mg^2+^ deficiency was defined as Mg^2+^ < 0.7 mmol/L, whereas Mg^2+^ levels ≥ 0.7 mmol/L were considered as sufficient using a single Mg^2+^ measurement. The estimated glomerular filtration rate (eGFR) was calculated using the CKD-EPI (Chronic Kidney Disease Epidemiology Collaboration) Creatinine Equation [21]. Serum levels of tacrolimus and cyclosporine A were collected within one week of Mg^2+^ measurements.

Pre-transplant diabetes mellitus (DM) was defined according to the American Diabetes Association (ADA) Guidelines [22] or as an intake of glucose-lowering drugs according to the patient records.

Delayed graft function was defined as acute kidney injury (AKI) that occurred in the first week of KT, which necessitated dialysis intervention [23].

### 2.3. Outcomes

The primary outcome of this study was the incidence of UTIs and viral infections (CMV, polyoma, and/or EBV) within one year after KT. Secondary outcomes were hospitalization due to infection, incidence rates of long-term (up to two years) infections, as well as all-cause mortality.

All-cause mortality data were requested from the database of the Main Association of Austrian Social Insurance Institutions (Hauptverband der österreichischen Sozialversicherungsträger, last accessed on 15 August 2019). Data on infection-related mortality during the first year after KT, including those with death due to sepsis, were also collected from OEDTR.

### 2.4. Statistical Analysis

Continuous parameters are summarized as the median and interquartile range (IQR), whereas categorical parameters are presented as absolute and relative frequencies. Differences between Mg^2+^ deficient and non-deficient patients were assessed either with the Mann–Whitney U or χ^2^ test. To identify factors associated with infections, a logistic regression analysis was performed using clinical and known risk factors and possible confounders (age at time of KT, sex, body mass index (BMI), dialysis modality (hemodialysis), dialysis vintage (<1 vs. ≥1 year), living kidney donation, donor and recipient CMV seropositivity, glomerulonephritis (GN) as primary renal disease, maintenance immunosuppression (defined as highest tertile of tacrolimus and cyclosporine A serum level at the time of the Mg^2+^ measurement), nicotine abuse, diabetes mellitus (DM), delayed graft function, eGFR, albumin, and previous KT). To further analyze the influence of Mg^2+^ deficiency on the incidence of infections, the analyses for Mg^2+^ deficiency were adjusted for all variables listed above. Additionally, logistic regression analyses were performed to identify possible risk factors leading to an Mg^2+^ deficiency and to test the influence of the induction therapy on infection risk. Kaplan–Meier and Cox proportional hazard regression analyses were performed to assess the influence of Mg^2+^ deficiency on overall mortality. Results are presented as either odds ratios (ORs, logistic regression) or hazard ratios (HRs, Cox regression) with the respective 95% confidence intervals (CIs). A *p*-value < 0.05 was considered statistically significant, and all analyses were performed using SAS version 9.4 (SAS Institute, Cary, NC, USA).

## 3. Results

### 3.1. Patients’ Characteristics and Laboratory Findings

The study population consisted of 376 KTRs. The majority of the patients (258, 68.6%) were male, and the median age was 52.0 years (IQR 41.0–62.0). Detailed patient characteristics and laboratory findings of the study population are shown in Table 1. The median Mg^2+^ concentration of the whole study population was 0.67 (IQR 0.61–0.74) mmol/L, measured after a median time of 29 days (IQR: 20–45) after KT. A total of 229 patients (60.9%) had magnesium deficiency with a median serum Mg^2+^ concentration of 0.63 (IQR 0.58–0.66) mmol/L. KTRs with a Mg^2+^ deficiency had significantly higher rates of donor CMV seropositivity than patients without a deficiency (median 51.8% vs. 39.0%, *p* = 0.017). Other than that, the study groups were similar regarding clinical characteristics, underlying kidney diseases, comorbidities, and medication (Table 1).

Data of clinical and laboratory variables were available in >95% of all study participants, except for bicarbonate, which was just available in n = 339 (90.2%).

Mg^2+^ sufficient patients had significantly worse kidney function based on creatinine and eGFR measurements (*p* = 0.001 and *p* = 0.009, respectively). A significant correlation between Mg^2+^ levels and eGFR (Pearson correlation coefficient r = −0.138, *p* = 0.008) was observed.

At the time of the Mg^2+^ measurement, most patients (88.0%) used tacrolimus. The percentage of patients with an Mg^2+^ deficiency was significantly higher in patients with higher tacrolimus levels (percentages in the respective tacrolimus tertials: 56.1% vs. 61.7% vs. 74.6%; *p* = 0.014).

In total, *n* = 52 (15.2%) KTRs received oral Mg^2+^ supplementation (Table 1). No statistically significant difference in Mg^2+^ supplementation between patients with and without Mg^2+^ deficiency was observed (*p* = 0.424).

### 3.2. Percentage and Incidence of Infections

During the first year after KT, UTI was observed in 204 (54.3%), CMV in 182 (48.4%), EBV in 96 (25.5%), and polyoma in 33 (8.8%) patients. In total, 236 (62.8%) patients had a viral infection. The incidences for all infection types were lower during the second year after KT: 69 (19.0%) patients had UTI, 31 (8.5%) CMV, 5 (1.4%) polyoma, and 10 (2.7%) EBV for a total of 41 (11.3%) patients with a viral infection.

There was significant difference in UTI incidence rates within the first 12 months between the patients with and without Mg^2+^ deficiency (58.5% vs. 47.6%, *p* = 0.039). Moreover, a significantly higher incidence rate of viral infections was observed in patients with Mg^2+^ deficiency in the first year after KT (69.9% vs. 51.7%, *p* < 0.001). There were significantly lower incidence rates of UTI and viral infections between the 12–24-month time period as compared to the first year after KT (McNemar test: both *p* < 0.001). However, no significant differences in the infection incidence rates 12–24 months post-transplant were observed between the study groups (*p* = 0.807 for UTI and *p* = 0.474 for viral infections). A total of 72 (19.1%) patients required hospital admission due to infection during the first year, and 27 (7.4%) in the second year after KT. The incidence rates of UTI and viral infections at the two time intervals after KT according to the study groups (Mg^2+^ < 0.7 mmol/L and Mg^2+^ levels ≥ 0.7 mmol/L) are shown in Table 2.

### 3.3. Risk Factors for Infections

Regarding Mg^2+^ deficiency, the univariable logistic regression analysis 12 months after KT revealed significant associations with both UTIs and viral infections compared to Mg^2+^ sufficiency (OR: 1.55, 95% CI: 1.02–2.35, *p* = 0.039 and OR: 2.17, 95% CI: 1.41–3.33, *p* < 0.001). Age at the time of KT, female sex, BMI, and delayed graft function were significantly associated with higher UTI incidence, while higher eGFR, living kidney donation, and serum albumin levels were associated with a decreased incidence of UTIs within 12 months after KT (Table S1). On the other hand, age at the time of KT and donor CMV serostatus showed a significant higher association with the incidence of viral infections, while a higher eGFR and serum albumin levels also showed an association with decreased viral infection incidence rates within 12 months after KT (Table S2).

In multivariable logistic regression analyses, including all parameters, serum Mg^2+^ deficiency remained a significant predictor of UTIs and viral infections during the first year after KT (OR: 1.73, 95% CI: 1.04–2.86, *p* = 0.035 and OR: 2.05, 95% CI: 1.23–3.41, *p* = 0.006, respectively) (Table 3).

In a subgroup analysis for patients receiving IL2Ra (*n* = 242), Mg^2+^ deficiency remained significantly associated with higher incidences of viral infections during the first year after KT (OR: 2.484, 95% CI: 1.28–4.80, *p* = 0.007) but not for UTIs (OR: 1.22, 95% CI: 0.66–2.27, *p* = 0.529). A similar analysis in patients receiving ATG (*n* = 50) did not show significant associations, likely due to the small number of observations.

### 3.4. Risk Factors for Magnesium Deficiency

In the univariable analysis, serum phosphorus level < 2.6 mg/dL (OR: 2.54, 95% CI: 1.64–3.93, *p* < 0.001), CRP levels > 5 mg/dL (OR: 0.61, 95% CI: 0.38–0.96, *p* = 0.035), high CNI serum levels (OR: 2.29, 95% CI: 1.35–3.89, *p* = 0.002 for comparison of highest vs. lowest tertial), and donor CMV seropositivity (OR: 1.68, 95% CI: 1.09–2.58, *p* = 0.018) were associated with the presence of Mg^2+^ deficiency. All factors investigated for the risk of hypomagnesemia are shown in Table S3.

### 3.5. Mortality

During the first year after transplantation, 12 (3.2%) patients died, five of which had an infection as a likely cause of death. During a median follow-up period of 6.7 (IQR 4.9–9.3) years, 67 (17.8%) patients died due to any cause. In Cox regression analysis, Mg^2+^ deficiency was not significantly associated with all-cause mortality (HR: 1.15, 95% CI: 0.70–1.89, *p* = 0.577; Figure S1). However, age at the time of the transplantation (HR: 1.08, 95% CI: 1.05–1.10, *p* < 0.001), BMI (HR: 1.07, 95% CI: 1.01–1.13, *p* = 0.017), DM (HR: 2.04, 95% CI: 1.18–3.50, *p* = 0.010), delayed graft function (HR: 1.88, 95% CI: 1.16–3.03, *p* = 0.010) and donor CMV seropositivity (HR: 1.66, 95% CI: 1.02–2.73, *p* = 0.044) were associated with an increased risk of all-cause mortality. In contrast, glomerulonephritis as primary renal disease (HR: 0.42, 95% CI: 0.23–0.76, *p* = 0.004), serum albumin level (HR: 0.57, 95% CI: 0.39–0.84, *p* = 0.005) and eGFR at the time of the Mg^2+^ measurement (HR: 0.98, 95% CI: 0.97–0.99, *p* = 0.002) were associated with decreased risk of all-cause mortality. Sex, maintenance immunosuppression, hemodialysis, living kidney donation, recipient CMV serostatus, and smoking status were not associated with an increased risk of all-cause mortality.

## 4. Discussion

Opportunistic infections are a common and significant cause of morbidity, graft loss, reduced quality of life, and mortality among KTRs [15,24,25]. Although the incidence of these infections is highest shortly after transplantation, these infections continue to have a significant impact on outcomes after this period. In this study, we observed Mg^2+^ deficiency in more than sixty percent of KTRs within three months post-transplant and found that Mg^2+^ deficiency is associated with an increased incidence of UTIs and viral infections within the first year after KT, independent of potential confounders.

Hypomagnesaemia is a known risk factor for new-onset DM after transplantation (NODAT) [26] and seems to play a role in cardiovascular (CV) morbidity and mortality after KT [27,28,29]. Moreover, there has been an increasing interest in the potential role of Mg^2+^ in the immune system and a possible regulatory function in acquired immunity by regulating the proliferation and development of lymphocytes [30]. Several early studies provided evidence on a close relationship between Mg^2+^ and the inflammatory response in animal models [31,32,33]. In a mouse model, reduced serum Mg^2+^ concentration led to impaired CD8^+^ T-cell response to influenza A virus infection, reduced T-cell activity, and exacerbated mortality [34]. In humans, intracellular free Mg^2+^ controls the expression of the activating receptor natural killer group 2 member D (NKG2D) and is required for the cytotoxic activity of NK and CD8^+^ T-cells [18]. Moreover, a case report suggested that in vitro addition of Mg^2+^ may restore the cytotoxicity of CD8^+^ T-cells in patients with mutations in interleukin-2-inducible T-cell kinase (ITK) and magnesium transporter 1 (MAGT1) [35]. Additionally, supplemental Mg^2+^ might also indirectly influence T-cell receptor signaling by binding several protein kinases [36].

Data from clinical studies on metabolic syndrome revealed a direct link between hypomagnesemia and inflammation, indicating its role in more complex inflammatory processes [37,38]. T-cell activation is an energy-dependent process driven by a switch from oxidative phosphorylation to aerobic glycolysis [39]. T-cells upregulate insulin receptors, which is necessary for their effective function [40]. Consequently, impaired insulin responsiveness may lead to impaired adaptive immunity. Several studies have revealed an association between hypomagnesemia and type 2 DM and NODAT [41,42], while oral Mg^2+^ supplementation increases insulin sensitivity and metabolic control in type 2 DM patients [37], but not in those with NODAT [43,44]. Importantly, DM as a cause of end-stage renal disease (ESRD) is associated with an increased risk of infectious death during the first post-transplant year in KTRs [45]. In our patient cohort, 15.4% of the patients had DM prior to KT, which was not a risk factor for UTIs or viral infections during the first year after KT. Nevertheless, insufficient glycemic control in KTRs might be an essential aspect of post-transplant infections since insulin plays a pivotal role in the activation of T-cells, and this link with Mg^2+^ should be explored further in mechanistic studies.

Recent clinical data revealed worse mortality rates in patients with pneumonia and hypomagnesemia admitted to the intensive care unit (ICU) [46]. In pediatric liver transplant recipients with pre-transplant hypomagnesemia, increased mortality risk due to sepsis was observed [47]. Despite the importance of opportunistic infections for outcomes in KTRs and the high prevalence of Mg^2+^ deficiency in this setting, to our knowledge, only one study has investigated the potential impact of Mg^2+^ on infection complications after KT. In their well-designed, single-center prospective cohort study, Van Laecke and colleagues investigated 873 KTRs and found a dose-dependent association between a single baseline serum Mg^2+^ concentration and incidence of severe infections in KTRs [19]. However, in our study, we mainly focused on mild transplant-related infections managed in the daily routine practice in the transplant out- and inpatient setting with different primary endpoints. Our findings reflect an observation in a cohort of KTRs managed in an ambulatory setting or admitted to the ward due to reasons primarily not necessarily associated with UTIs or viral infections. This is an important aspect since an efficient strategy to prevent severe infections after KT is required. Moreover, apart from severe infections, such as CMV viral syndrome and tissue invasive disease, a number of indirect immunomodulatory effects of viral infections on long-term kidney function have been postulated [48]. This indirect connection may lead to an increased incidence of acute and chronic rejection after KT, which may be caused by a bystander activation of alloreactive T-cells during an antiviral response of the organ recipient. Additionally, the incidence of other opportunistic infections may also be influenced by these effects [48]. Nevertheless, the results of these studies support each other regarding the observation between Mg^2+^ deficiency and infections among KTRs and provide additional evidence on the role of Mg^2+^ on infections in an independent cohort of KTRs.

Current immunosuppression strategies block T-lymphocytes primarily to prevent cellular rejection. The use of mTORi among KTRs seems to be associated with a reduced risk of CMV infections compared to those treated with a regular dose of CNI alone. Moreover, a combination of mTORi and a reduced dose of CNI also revealed the same effect. Interestingly, polyoma infections were not influenced by the different immunosuppression regimens [49,50]. In our cohort, Mg^2+^ deficiency remained an independent risk factor of UTIs and viral infections in the univariable analysis and after adjustment for possible confounders (even including maintenance immunosuppression therapy). However, the percentage of Mg^2+^ deficiency was higher in patients with the highest tertile of serum tacrolimus concentrations. Adverse events of CNIs include renal Mg^2+^ wasting leading to Mg^2+^ deficiency [51]. Thus, CNIs might indirectly further increase the susceptibility of KTRs for viral infections. On the contrary, CNI avoidance and withdrawal might lead to acute graft rejection, while a reduced dose of CNIs (particularly low-dose tacrolimus regimen in combination with an interleukin (IL)-2 receptor blocker) in induction regimes seems to be appropriate to reduce acute rejection [49,52]. Since T-cells are responsible for controlling viral infections and a direct link between T-cell function and Mg^2+^ exists, a comprehensive approach investigating the associations between immunosuppression, Mg^2+^, and particularly viral infections after KT is warranted.

Importantly, there was also a significant difference in the incidence of UTIs during the first year after KT between the patients with and without Mg^2+^ deficiency. This observation might also be in line with the present knowledge on the effect of Mg^2+^ on the immune function, significantly affecting the NK and CD8+ T-cell function [7,18]. Basically, in response to UTIs, a wide range of cells of the innate immune system, such as neutrophils, macrophages, and mast cells, are involved. The possible role of NK cells on UTIs (or bacterial infections) via tumor necrosis factor (TNF) production was reported [53]. However, their exact role in the pathogenesis of UTIs remains unclear. In addition, adaptive immune responses seem to be also limited in the immune response for UTIs [54].

In our cohort, patients with Mg^2+^ deficiency had lower serum phosphate levels and better kidney function compared with patients without Mg^2+^ deficiency. Hypophosphatemia is a common finding in KTRs, especially in those with immediate graft function and a high pre-transplant serum PTH level due to the significant urinary phosphorus loss driven by the effects of high levels of PTH and FGF-23 [55]. The constellation of lower serum Mg^2+^ and phosphate levels may be a marker of a better tubular graft function. Nevertheless, serum phosphate levels start to normalize within the first few months after KT due to the reduced FGF-23 levels [56], while hypomagnesemia might persist for several years after KT. The relationship between decreased serum Mg^2+^ levels and accelerated graft function decline or development of renal lesions involving innate immune pathways has been discussed [42]. However—until now—no clear association for these relationships was found. Dietary and supplementary interventions containing Mg^2+^ and phosphate may lead to better nutritional status and indirectly improve immune function, especially within the first year after KT. However, prospective studies on this issue are needed.

Hypomagnesaemia is a known predictor of CV and all-cause mortality in dialysis patients [17,57,58]. Among KTRs, a possible relationship between the accelerated decline of graft function and hypomagnesemia was suggested [42]. Garnier and colleagues hypothesized an indirect positive effect of Mg^2+^ on CV-related morbidity and mortality through decreased CV risk as a beneficial effect of Mg^2+^ supplementation [42]. To our knowledge, data on long-term all-cause mortality and Mg^2+^ status among KTRs are lacking. In our analysis, no statistically significant association between baseline serum Mg^2+^ concentration and all-cause mortality was observed.

Some limitations should be considered when interpreting the results. First, given the design of the study as a single-center analysis based on retrospectively collected data, missing data were unavoidable. In the early years of the observation period, CMV PCR testing was not widely available, and a pp65 antigenemia assay was frequently used to identify CMV infections. However, in this period, this semi-quantitative fluorescent assay based on the detection of CMV infected cells in peripheral blood was the standard diagnostic approach to identify CMV infections in KTRs [59]. Notably, this assay is comparable in sensitivity to CMV PCR [60]. Second, induction therapies might represent an essential aspect of incidence rates of opportunistic infections after KT [61,62]. Good quality systematic review data provided clear evidence on the increased risk of CMV infections in patients treated by ATG [63]. In our subgroup analysis, Mg^2+^ deficiency was significantly associated with higher incidence of viral infections, but not with UTIs during the first year after KT in patients receiving IL2Ra in a multivariate analysis. This might be explained due to the high incidence rate of UTIs in the first 6 months after KT, which is a time period with a higher effect rate of IL2Ra. This result may allude to the significant effect of IL2Ra on UTIs. In contrast, a similar analysis in patients receiving ATG did not show these results in our patient cohort. However, these observations need to be interpreted with caution due to small number of observations (n = 50 in the ATG group) as well as missing data, and a possible link should be investigated more extensively. On the other hand, recent evidence shows the decline of infection risk in KTRs that received lower ATG doses [64]. Additionally, previous data suggested no influence of induction therapy on severe infections among KTRs with hypomagnesemia [19]. Third, most Mg^2+^ is found intracellular, and only around 1% is present in the blood, representing a small fraction of the total body reserves. Thus, serum Mg^2+^ concentration may not represent intracellular Mg^2+^ availability, which is an overall limitation on studies interpreting data using serum Mg^2+^ measurements. Current methods estimating intracellular Mg^2+^ concentration are invasive and expensive with low evidence level on their efficacy. Nevertheless, serum Mg^2+^ measurement is the most available and commonly used test to access Mg^2+^ status [65]. In addition, in blood, 20–30% of Mg^2+^ is bound to albumin and other serum proteins. In our cohort, only a small proportion of KTRs (24.3%) had a serum albumin level < 3.5 g/dL. Additionally, serum Mg^2+^ concentration can be influenced by many factors, including pH, azotemia, insulin resistance, post-transplantation volume expansion, low dietary Mg^2+^ intake, or time of blood sample taken [42,66]. In this analysis, we used a single Mg^2+^ measurement, and the question arises as to the variability within an individual patient. However, hypomagnesemia is an extensively described phenomenon in KTRs due to several pathophysiological and clinical factors [9,42], and it is rather unlikely that these factors potentially move patients by one to another study group. Finally, we do not have data on proton-pump inhibitor (PPI) therapies, which are a possible risk factor for hypomagnesemia and frequently prescribed for KTRs [67]. The possible association between the use of PPIs and Mg^2+^ in link with infection complications should be addressed in future studies. Nevertheless, to the best of our knowledge, this is the first study addressing the impact of serum Mg^2+^ on opportunistic infections and UTIs among KTRs managed in an ambulatory setting or admitted to the ward due to reasons not associated with infection-related complications.

## 5. Conclusions

In our study involving KTRs, Mg^2+^ deficiency was independently associated with UTIs and viral infections in the early phase after KT. Our findings have implications for both research and clinical practice. The independent association between Mg^2+^ deficiency and UTIs and viral infections highlights the need to explore the immunological effects of Mg^2+^ in KTRs in more detail. Specific risk factors for Mg^2+^ deficiency, particularly different immunosuppressive strategies, may further be characterized and used for more intensive serum Mg^2+^ controlling to improve its potential effects not only on infection risk but on CV risk factors as well. In clinical praxis, the critical evaluation and potential use of reduced CNI regimes and correction of serum Mg^2+^ level might be beneficial to preventing viral infections among KTRs. The current results support the hypothesis that Mg^2+^ plays an important role in adaptive immunity among KTRs. Further, especially interventional studies on Mg^2+^ and opportunistic infections in KTRs are warranted, at best designed as randomized placebo-controlled trials on Mg^2+^ supplementation in KTRs with Mg^2+^ deficiency.

## Figures and Tables

**Table 1:** Baseline patient characteristics and laboratory results in the whole study population and stratified according to the presence and absence of Mg^2+^ deficiency.

	Whole Study Population (*N* = 376)	Patients without Mg^2+^ Deficiency (*N* = 147)	Patients with Mg^2+^ Deficiency (*N* = 229)	*p*-Value
**Age at time of KT (years)**	52.0 (41.0–62.0)	50.0 (40.0–61.0)	53.0 (42.0–62.0)	0.133
**Gender (female)**	118 (31.4)	41 (27.9)	77 (33.6)	0.242
**BMI (kg/m^2^)**	24.9 (22.1–28.1)	24.4 (22.1–28.1)	25.2 (22.3–28.0)	0.372
**Nicotine abuse**	169 (44.9)	71 (48.3)	98 (42.8)	0.295
**Dialysis-related data**				
Hemodialysis	293 (77.9)	114 (77.6)	179 (78.2)	0.888
Peritoneal dialysis	62 (16.5)	25 (17.0)	37 (16.2)	0.829
Preemptive KT	21 (5.6)	8 (5.4)	13 (5.7)	0.923
Dialysis vintage (months)	40.5 (22.0–71.0)	35.0 (18.0–58.0)	42.0 (22.0–75.0)	0.208
**Comorbidities**				
Diabetes mellitus	58 (15.4)	28 (19.0)	30 (13.1)	0.119
Dyslipidemia	197 (52.4)	75 (51.0)	122 (53.3)	0.669
Hypertension	357 (94.9)	138 (93.9)	219 (95.6)	0.448
Coronary heart disease	37 (9.8)	16 (10.9)	21 (9.2)	0.586
**Transplantation-related data**				
Living kidney donation	47 (12.5)	19 (12.9)	28 (12.2)	0.842
Previous KT	80 (21.3)	27 (18.4)	53 (23.1)	0.269
Donor CMV seropositivity	170 (46.8)	55 (39.0)	115 (51.8)	0.017
Recipient CMV seropositivity	225 (61.8)	85 (59.9)	140 (63.1)	0.539
GN as primary renal disease	130 (34.6)	50 (34.0)	80 (34.9)	0.855
Delayed graft function	133 (35.4)	56 (38.1)	77 (33.6)	0.376
**Immunosuppression**				
CNI	374 (99.5)	145 (98.6)	229 (100)	0.077
mTOR inhibitor	3 (0.8)	2 (1.4)	1 (0.4)	0.326
Antiproliferative agents	372 (98.9)	144 (98.0)	228 (99.6)	0.139
Laboratory data *				
Leukocytes (10 × 9/L)	8.3 (6.3–10.7)	8.7 (6.4–10.6)	8.1 (6.3–10.7)	0.471
C-reactive protein (mg/dL)	2.7 (1.0–5.9)	2.8 (1.0–7.2)	2.4 (1.0–4.9)	0.151
Parathyroid hormone (pg/mL)	155.2 (105.8–234.8)	155.7 (94.6–237.1)	153.6 (108.5–228.7)	0.644
Calcium, total (mmol/L)	2.4 (2.3–2.5)	2.4 (2.3–2.5)	2.4 (2.3–2.5)	0.239
Phosphate (mg/dL)	2.3 (1.9–2.9)	2.5 (2.0–3.3)	2.2 (1.7–2.7)	<0.001
Bicarbonate (mmol/L)	22.1 (20.1–24.5)	22.2 (19.9–24.9)	22.0 (20.3–24.2)	0.594
Creatinine (mg/dL)	1.5 (1.3–1.9)	1.7 (1.3–2.3)	1.5 (1.3–1.8)	0.001
eGFR (ml/min/1.73 m^2^)	47.0 (35.5–59.8)	41.7 (31.0–59.6)	49.2 (38.4–60.4)	0.009
Albumin (g/dL)	3.9 (3.5–4.3)	3.8 (3.4–4.4)	3.9 (3.5–4.3)	0.950
25(OH)-vitamin D (ng/mL)	23.7 (15.2–31.4)	21.1 (14.0–29.2)	25.2 (17.3–32.8)	0.007
**Magnesium supplementation**	57 (15.2)	25 (17.0)	32 (14.0)	0.424

Statistically significant *p*-values appear in boldface type (*p* < 0.05). Continuous variables are expressed as median (25th to 75th percentile). Categorical variables are n (%). * Laboratory data at time of Mg^2+^ measurement. Subsections appear in boldface type. Abbreviations: BMI: body mass index, CMV: cytomegalovirus, CNI: calcineurin inhibitor, eGFR: estimated glomerular filtration rate, GN: glomerulonephritis, KT: kidney transplantation, mTOR: mammalian target of rapamycin.

**Table 2:** Incidence rate of all urinary tract and viral infections during the first and second year after KT.

Type of Infections	Whole Study Population		Patients without Mg^2+^ Deficiency		Patients with Mg^2+^ Deficiency	
	*N* = 376	*N* = 364	*N* = 147	*N* = 143	*N* = 229	*N* = 221
	0–12 Months	12–24 Months	0–12 Months	12–24 Months	0–12 Months	12–24 Months
Urinary tract infections	204 (54.3)	69 (19.0)	70 (47.6)	28 (19.6)	134 (58.5)	41 (18.6)
Viral infections	236 (62.8)	41 (11.3)	76 (51.7)	14 (9.8)	160 (69.9)	27 (12.2)
Detailed viral infections						
CMV	182 (48.4)	31 (8.5)	57 (38.8)	10 (7.0)	125 (54.6)	21 (9.5)
Polyoma	33 (8.8)	5 (1.4)	8 (5.4)	1 (0.7)	25 (10.9)	4 (1.8)
EBV	96 (25.5)	10 (2.7)	37 (25.2)	4 (2.8)	59 (25.8)	6 (2.7)

Categorical variables are n (%). Abbreviations: CMV: cytomegalovirus, EBV: Epstein–Barr virus.

**Table 3:** Multivariable logistic regression analysis of confounders for urinary tract and viral infections incidence during the first year after KT.

Test Variable	Urinary Tract Infections			Viral Infections		
	OR	95% CI	*p*-Value	OR	95% CI	*p*-Value
Age at the time of KT (years)	1.01	0.99–1.04	0.179	1.00	0.98–1.02	0.982
Gender (female)	**4.57**	**2.56–8.16**	**<0.001**	1.14	0.66–1.98	0.635
BMI (kg/m^2^)	1.06	0.99–1.13	0.090	1.05	0.98–1.12	0.179
Nicotine abuse	1.36	0.83–2.22	0.217	1.65	1.00–2.74	0.051
Serum Mg^2+^ (deficiency)	**1.73**	**1.04–2.86**	**0.035**	**2.05**	**1.23–3.41**	**0.006**
eGFR	1.00	0.98–1.01	0.603	0.99	0.98–1.00	0.150
Albumin	0.74	0.49–1.12	0.155	0.73	0.48–1.12	0.146
CNI serum level (highest tertile)	0.98	0.59–1.63	0.940	1.68	0.98–2.86	0.059
Dialysis vintage (<1 year)	1.06	0.43–2.59	0.898	0.66	0.27–1.61	0.362
Hemodialysis	0.85	0.47–1.53	0.587	0.91	0.50–1.66	0.760
Previous KT	0.84	0.46–1.54	0.573	0.88	0.47–1.64	0.681
Living kidney donation	0.53	0.21–1.38	0.194	1.57	0.61–4.07	0.349
Donor CMV seropositivity	1.12	0.69–1.82	0.650	**2.54**	**1.54–4.18**	**<0.001**
Recipient CMV seropositivity	0.67	0.41–1.11	0.123	1.66	1.00–2.76	0.051
Delayed graft function	1.47	0.83–2.60	0.190	1.19	0.97–2.13	0.552
Diabetes mellitus	1.06	0.52–2.18	0.877	0.82	0.40–1.69	0.589
GN as primary kidney disease	0.72	0.44–1.20	0.205	0.80	0.48–1.34	0.387

Statistically significant *p*-values appear in boldface type (*p* < 0.05). Abbreviations: BMI: body mass index, CI: confidence interval; CMV: cytomegalovirus, CNI: calcineurin inhibitor, eGFR: estimated glomerular filtration rate, GN: glomerulonephritis, KT: kidney transplantation, mTOR: mammalian target of rapamycin, OR: odds ratio.

## Data Availability

The data presented in this study are available from the corresponding author on reasonable request.

## Supplementary Materials

The following are available online at https://www.mdpi.com/article/10.3390/nu13041296/s1, Figure S1: All-cause mortality comparing patients with a serum Mg^2+^ level ≥ 0.7 mmol/L and < 0.7 mmol/L, Table S1: Unadjusted logistic regression analysis of risk factors for hypomagnesemia, Table S2: Unadjusted logistic regression analysis of risk factors for viral infections, Table S3: Unadjusted logistic regression analysis of risk factors for hypomagnesemia.
