# Supplementary material for: Hypomagnesemia Is a Risk Factor for Infections after Kidney Transplantation: A Retrospective Cohort Analysis
Source: Nutrients. 2021 Apr 14;13(4):1296. doi: 10.3390/nu13041296 (PMC8070921; doi:10.3390/nu13041296)
Supplement: Supplementary file 1 [file nutrients-13-01296-s001.zip › nutrients-1173439-supplementary/nutrients-1173439-Table s-3.docx]

**Table S1.** Unadjusted logistic regression analysis of risk factors for UTI

|  | **Unadjusted logistic regression** | | | | |
| --- | --- | --- | --- | --- | --- |
|  | **No UTI** | **UTI** | OR | 95%CI | p-value |
| Age at the time of KT (years) | **48.0 (39.0-58.0)** | **54.0 (43.0-64.0)** | **1.02** | **(1.01-1.04)** | **0.002** |
| Gender (female) | **31 (18.0)** | **87 (42.6)** | **3.38** | **(2.10-5.45)** | **<0.001** |
| BMI (kg/m2) | **24.4 (21.5-27.4)** | **25.4 (23.1-28.7)** | **1.07** | **(1.02-1.13)** | **0.006** |
| Nicotine abuse | 78 (45.3) | 91 (44.6) | 0.97 | (0.65-1.46) | 0.886 |
| Serum Mg^2+^ (deficiency) | **95 (55.2)** | **134 (65.7)** | **1.55** | **(1.02-2.35)** | **0.039** |
| eGFR | **52.4 (38.1-63.8)** | **44.1 (32.1-57.2)** | **0.98** | **(0.97-0.99)** | **<0.001** |
| Albumin | **4.0 (3.5-4.4)** | **3.8 (3.4-4.2)** | **0.63** | **(0.45-0.89)** | **0.009** |
| CNI serum level (highest tertial) | 59 (34.7) | 65 (32.3) | 0.90 | (0.58-1.39) | 0.630 |
| Hemodialysis | 133 (77.3) | 160 (78.4) | 1.07 | (0.65-1.74) | 0.797 |
| Peritoneal dialysis | 28 (16.3) | 34 (16.7) | 1.03 | (0.60-1.78) | 0.920 |
| Previous KT | 42 (24.4) | 38 (18.6) | 0.71 | (0.43-1.16) | 0.173 |
| Living Kidney Donation | **28 (16.3)** | **19 (9.3)** | **0.53** | **(0.28-0.98)** | **0.044** |
| Donor CMV seropositivity | 71 (43.8) | 99 (49.3) | 1.24 | (0.82-1.89) | 0.303 |
| Recipient CMV seropositivity | 103 (62.4) | 122 (61.3) | 0.95 | (0.62-1.46) | 0.827 |
| Delayed graft function | **51 (29.7)** | **82 (40.2)** | **1.59** | **(1.04-2.45)** | **0.034** |
| Diabetes mellitus | 22 (12.8) | 36 (17.6) | 1.46 | (0.82-2.59) | 0.196 |
| GN as primary kidney disease | 66 (38.4) | 64 (31.4) | 0.73 | (0.48-1.12) | 0.156 |

Statistically significant P values appear in boldface type (P < 0.05). Continuous variables are expressed as median (25th to 75th percentile). Categorical variables are n (%).

**Abbreviations:** BMI: body mass index, CI: confidence interval; CMV: cytomegalovirus, CNI: calcineurin inhibitor, eGFR: estimated glomerular filtration rate, GN: glomerulonephritis, KT: kidney transplantation, Mg^2+^: magnesium OR: odds ratio

**Table S2.** Unadjusted logistic regression analysis of risk factors for viral infections

|  | **Unadjusted logistic regression** | | | | |
| --- | --- | --- | --- | --- | --- |
|  | **No viral infections** | **viral infections** | OR | 95%CI | p-value |
| Age at the time of KT (years) | **48.5 (40.0-58.0)** | **53.5 (42.0-64.0)** | **1.02** | **(1.00-1.03)** | **0.047** |
| Gender (female) | 42 (30.0) | 76 (32.2) | 1.11 | (0.70-1.74) | 0.656 |
| BMI (kg/m2) | 24.8 (22.2-26.9) | 24.9 (22.1-28.7) | 1.05 | (1.00-1.10) | 0.073 |
| Nicotine abuse | 54 (38.6) | 115 (48.7) | 1.51 | (0.99-2.32) | 0.056 |
| Serum Mg2+ (deficiency) | **69 (49.3)** | **160 (67.8)** | **2.17** | **(1.41-3.33)** | **<0.001** |
| eGFR | **51.7 (38.4-62.5)** | **44.7 (32.8-59.0)** | **0.99** | **(0.98-1.00)** | **0.025** |
| Albumin | **4.0 (3.6-4.4)** | **3.8 (3.4-4.3)** | **0.66** | **(0.46-0.94)** | **0.022** |
| CNI serum level (highest tertial) | 38 (27.5) | 86 (36.9) | 1.54 | (0.97-2.44) | 0.065 |
| Hemodialysis | 107 (76.4) | 186 (78.8) | 1.15 | (0.70-1.89) | 0.590 |
| Peritoneal dialysis | 24 (17.1) | 38 (16.1) | 0.93 | (0.53-1.62) | 0.793 |
| Previous KT | 31 (22.1) | 49 (20.8) | 0.92 | (0.55-1.53) | 0.752 |
| Living Kidney Donation | 19 (13.6) | 28 (11.9) | 0.86 | (0.46-1.60) | 0.629 |
| Donor CMV seropositivity | **42 (31.8)** | **128 (55.4)** | **2.66** | **(1.70-4.17)** | **<0.001** |
| Recipient CMV seropositivity | 77 (56.2) | 148 (65.2) | 1.46 | (0.95-2.25) | 0.088 |
| Delayed graft function | 48 (34.3) | 85 (36.0) | 1.08 | (0.70-1.67) | 0.734 |
| Diabetes mellitus | 21 (15.0) | 37 (15.7) | 1.05 | (0.59-1.88) | 0.861 |
| GN as primary kidney disease | 53 (37.9) | 77 (32.6) | 0.79 | (0.51-1.23) | 0.303 |

Statistically significant P values appear in boldface type (P < 0.05). Continuous variables are expressed as median (25th to 75th percentile). Categorical variables are n (%).

**Abbreviations:** BMI: body mass index, CI: confidence interval; CMV: cytomegalovirus, CNI: calcineurin inhibitor, eGFR: estimated glomerular filtration rate, GN: glomerulonephritis, KT: kidney transplantation, Mg^2+^: magnesium OR: odds ratio

**Table S3.** Unadjusted logistic regression analysis of risk factors for hypomagnesemia

| **Test variable** | **Unadjusted logistic regression** | | | | |
| --- | --- | --- | --- | --- | --- |
|  | **Patients without Mg^2+^ deficiency**  **(N=147)** | **Patients with Mg^2+^ deficiency**  **(N=229)** | OR | 95% CI | p-value |
| Age at the time of KT (years) | 50.0 (40.0-61.0) | 53.0 (42.0-62.0) | 1.01 | (1.00-1.03) | 0.059 |
| Gender (female) | 41 (27.9) | 77 (33.6) | 1.31 | (0.83-2.06) | 0.243 |
| BMI  (kg/m^2^) | 24.4 (22.1-28.1) | 25.2 (22.3-28.0) | 1.02 | (0.97-1.07) | 0.553 |
| Nicotine abuse | 71 (48.3) | 98 (42.8) | 0.80 | (0.53-1.21) | 0.295 |
| eGFR | 41.7 (31.0-59.6) | 49.2 (38.4-60.4) | 1.01 | (1.00-1.02) | 0.051 |
| Creatinine (> 1.2 mg/dL) | 119 (81.5) | 183 (79.9) | 0.90 | (0.53-1.53) | 0.704 |
| Calcium, total  (> 2.65 mmol/L) | 12 (8.4) | 26 (11.4) | 1.40 | (0.68-2.87) | 0.361 |
| Bicarbonat (< 22 mmol/L) | 60 (45.8) | 103 (49.5) | 1.16 | (0.75-1.80) | 0.505 |
| Phosphorus (< 2.6 mg/dL) | **76 (52.1)** | **168 (73.4)** | **2.54** | **(1.64-3.93)** | **<0.001** |
| Parathormone  (> 65 pg/mL) | 124 (89.2) | 208 (91.6) | 1.32 | (0.65-2.70) | 0.440 |
| C-reactive protein  (> 5 mg/dL) | **49 (34.0)** | **54 (23.9)** | **0.61** | **(0.38-0.96)** | **0.035** |
| CNI serum level |  | | | | |
| Highest tertile | **34 (23.6)** | **90 (39.6)** | **2.29** | **(1.35-3.89)** | **0.002** |
| Middle tertile | 53 (36.8) | 71 (31.3) | 1.16 | (0.70-1.91) | 0.569 |
| Hemodialysis | 114 (77.6) | 179 (78.2) | 1.04 | (0.63-1.71) | 0.888 |
| Peritoneal dialysis | 25 (17.0) | 37 (16.2) | 0.94 | (0.54-1.64) | 0.828 |
| Previous KT | 8 (5.4) | 13 (5.7) | 1.05 | (0.42-2.59) | 0.923 |
| Living Kidney Donation | 19 (12.9) | 28 (12.2) | 0.94 | (0.50-1.75) | 0.841 |
| Donor CMV seropositivity | **55 (39.0)** | **115 (51.8)** | **1.68** | **(1.09-2.58)** | **0.018** |
| Recipient CMV seropositivity | 85 (59.9) | 140 (63.1) | 1.14 | (0.74-1.76) | 0.540 |
| Delayed graft function | 56 (38.1) | 77 (33.6) | 0.82 | (0.53-1.27) | 0.377 |
| Diabetes mellitus | 28 (19.0) | 30 (13.1) | 0.64 | (0.36-1.12) | 0.121 |
| GN as primary kidney disease | 50 (34.0) | 80 (34.9) | 1.04 | (0.67-1.61) | 0.855 |

Statistically significant P values appear in boldface type (P < 0.05). Continuous variables are expressed as median (25th to 75th percentile). Categorical variables are n (%).

**Abbreviations:** BMI: body mass index, CI: confidence interval; CMV: cytomegalovirus, CNI: calcineurin inhibitor, eGFR: estimated glomerular filtration rate, GN: glomerulonephritis, KT: kidney transplantation, Mg^2+^: magnesium, OR: odds ratio
