# Supplementary figures and images for: Hypomagnesemia Is a Risk Factor for Infections after Kidney Transplantation: A Retrospective Cohort Analysis
Source: Nutrients. 2021 Apr 14;13(4):1296. doi: 10.3390/nu13041296 (PMC8070921; doi:10.3390/nu13041296)

**Figure S1.** All-cause mortality comparing patients with a serum  $\text{Mg}^{2+}$  level  $\geq 0.7$  mmol/L and  $< 0.7$  mmol/L

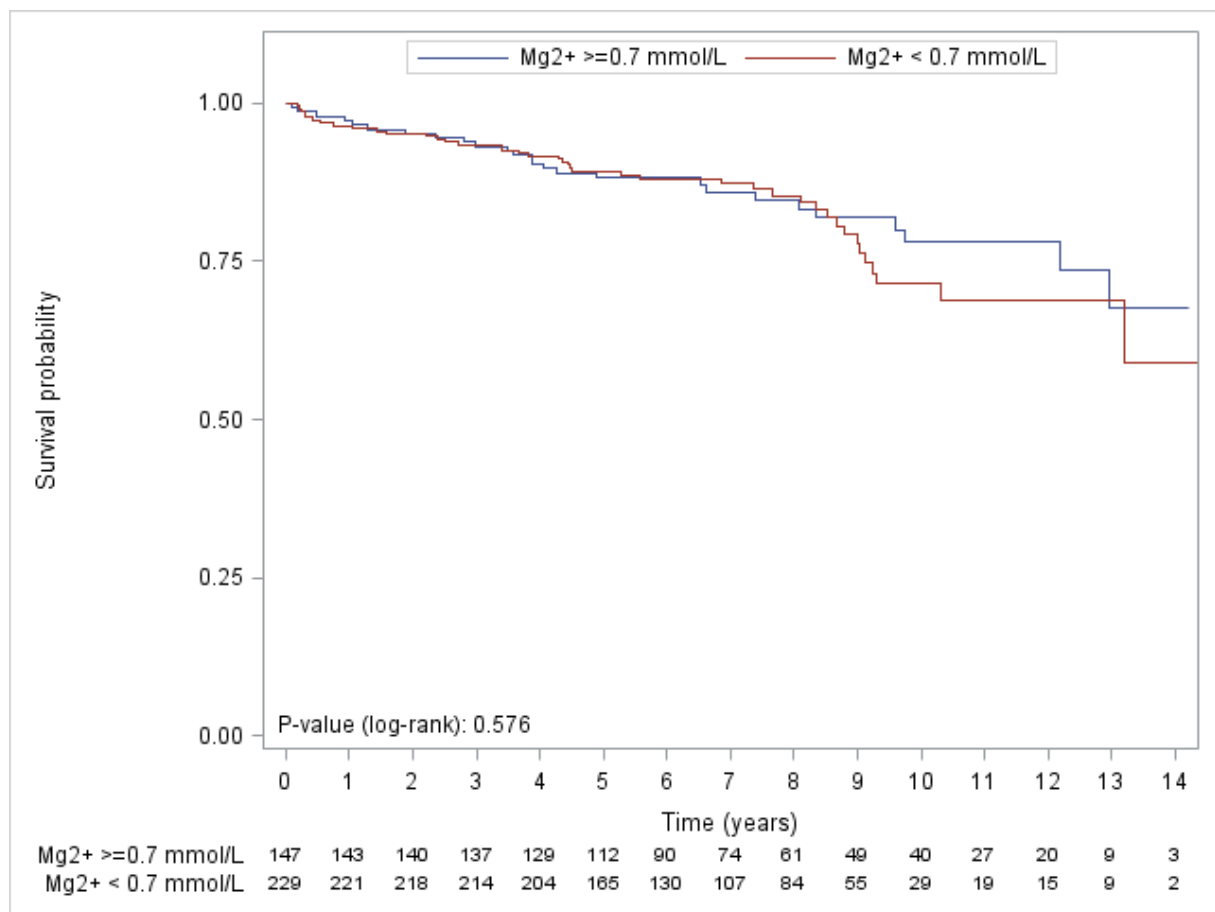

Supplement: Supplementary file 1 [file nutrients-13-01296-s001.zip › nutrients-1173439-supplementary/figure S1.pdf]
